# 3D Patterning of Si by Contact Etching With Nanoporous Metals

**DOI:** 10.3389/fchem.2019.00256

**Published:** 2019-04-25

**Authors:** Stéphane Bastide, Encarnacion Torralba, Mathieu Halbwax, Sylvain Le Gall, Elias Mpogui, Christine Cachet-Vivier, Vincent Magnin, Joseph Harari, Dmitri Yarekha, Jean-Pierre Vilcot

**Affiliations:** ^1^Institut de Chimie et des Matériaux Paris-Est (UMR 7182), CNRS, UPEC, Université Paris Est, Thiais, France; ^2^Institut d'Électronique de Microélectronique et de Nanotechnologie (IEMN), UMR 8520, Université de Lille, Villeneuve d'Ascq, France; ^3^Group of Electrical Engineering of Paris (GeePs), CNRS, Centralesupelec, Univ. Paris-Sud, Sorbonne Université, Gif sur Yvette, France

**Keywords:** silicon, nanoporous gold, imprinting, MACE, contact etching, patterning

## Abstract

Nanoporous gold and platinum electrodes are used to pattern n-type silicon by contact etching at the macroscopic scale. This type of electrode has the advantage of forming nanocontacts between silicon, the metal and the electrolyte as in classical metal assisted chemical etching while ensuring electrolyte transport to and from the interface through the electrode. Nanoporous gold electrodes with two types of nanostructures, fine and coarse (average ligament widths of ~30 and 100 nm, respectively) have been elaborated and tested. Patterns consisting in networks of square-based pyramids (10 × 10 μm^2^ base × 7 μm height) and U-shaped lines (2, 5, and 10 μm width × 10 μm height × 4 μm interspacing) are imprinted by both electrochemical and chemical (HF-H_2_O_2_) contact etching. A complete pattern transfer of pyramids is achieved with coarse nanoporous gold in both contact etching modes, at a rate of ~0.35 μm min^−1^. Under the same etching conditions, U-shaped line were only partially imprinted. The surface state after imprinting presents various defects such as craters, pores or porous silicon. Small walls are sometimes obtained due to imprinting of the details of the coarse gold nanostructure. We establish that np-Au electrodes can be turned into “np-Pt” electrodes by simply sputtering a thin platinum layer (5 nm) on the etching (catalytic) side of the electrode. Imprinting with np Au/Pt slightly improves the pattern transfer resolution. 2D numerical simulations of the valence band modulation at the Au/Si/electrolyte interfaces are carried out to explain the localized aspect of contact etching of n-type silicon with gold and platinum and the different surface state obtained after patterning. They show that n-type silicon in contact with gold or platinum is in inversion regime, with holes under the metal (within 3 nm). Etching under moderate anodic polarization corresponds to a quasi 2D hole transfer over a few nanometers in the inversion layer between adjacent metal and electrolyte contacts and is therefore very localized around metal contacts.

## Introduction

Metal Assisted Chemical Etching (MACE) of silicon began in the mid-2000s. Its new remarkable characteristic was to allow a localized dissolution of silicon around metal catalysts whose typical size is in the range of 10–100 nm. Due to the possibility of easily forming silicon nanowires and mesopores in crystalline silicon substrates, MACE has aroused considerable interest among the scientific community. Substantial efforts have been devoted to study the effects related to the nature of the metal catalyst and oxidizing agent, the crystal orientation of the silicon substrate and the concentration ratio “hydrofluoric acid/ oxidizing agent” (ρ) (Huang et al., 2011), as well as to produce ordered structures using films/metal grids of a larger characteristic size, typically a network of pillars of diameter and inter-distance of a few hundreds of nanometers (Huang et al., 2007).

More recently, a new research axis has developed around the imprinting of 3D structures in silicon by contact etching, based on the principles of MACE. *Imprinting* means that a macroscopic tool with the pattern to be transferred is brought into contact with the silicon substrate and removed, and can be reused several times. This tool is at least partly metallic in order to catalyze the dissolution of silicon in HF medium, with as oxidizing agent either a chemical species in solution or an anodic polarization using a potentiostat. This approach was first reported by the group of Kobayashi in 2011. Pattern transfer in (100) and (111) oriented c-Si was performed using as imprinting tool a NaOH texturized (100) c-Si substrate, (i.e., with square-based pyramids at the surface) covered by a SiNx layer and a platinum layer on the top acting as catalyst (Fukushima et al., 2011). The SiNx layer was meant to protect the silicon substrate of the etching tool to be self-etched by the platinum layer. Although the extent and quality of the pattern transfer appeared limited, inverted pyramids could be etched in (111) c-Si. This represented a remarkable achievement considering that this crystallographic orientation is incompatible with forming such structures.

Azeredo et al. reported on pattern transfer in porous silicon by contact etching in HF-H_2_O_2_ using macroscopic gold metallized pre-patterned stamps with a sinusoidal shape (Azeredo et al., 2016b). The shape transfer was impossible in silicon but easily obtained in porous silicon since the electrolyte could reach the metal interface through the porous silicon network. For silicon, this problem was partially alleviated by patterning with a 2.5D sinusoidal wave with sub-microscale dimensions (Azeredo et al., 2016a). The technique used a Cr/Au coated polyethylene sheet with a holographic surface (1 μm pitch and 350 nm amplitude) rolled around a platinum rod, immersed into HF-H_2_O_2_ and pressed on silicon with a load of 9 N. This process achieved a millimeter-scale parallel patterning with sub-100 nm resolution with a mirror-finish quality. Problems pointed out by the authors were the limited etch rates (imprinting time of 10 min) and some porosification of the silicon wafer (from a few nanometers to a few hundred nanometers from center to edge). Regrading etch rate, the work of Azeredo et al. (2016b) has clearly demonstrated that imprinting is a process mainly determined by the mass-transport of reactants and products. The overall etch rate dependence on the local depletion of reactant is characterized by quantitative means for the first time, which reveals the importance of the volume of reactant initially confined between the stamp and the substrate.

An electrochemical nanoimprint lithography (ECNL) approach based on MACE was also developed in the group of Zhan, for GaAs (Zhang et al., 2017a,b) and for silicon (Zhan et al., 2017). A review on electrochemical and nanomachining including ECNL is given in Zhan et al. (2017). In the case of silicon, a platinum metallized PDMS mold with a nanopillar array was used to imprint a nanohole array in (111) c-Si. Diameter and height of nanopillars were ~350 and 544 nm, respectively, and the imprinted nanoholes depth ~116 nm. The reason for incomplete in-depth imprinting was also attributed to the consumption and blocked mass transfer of reactant and etching products in the ultrathin electrolyte layer between the platinum metallized imprint mold and the silicon wafer. Using metal catalyst-coated grayscale stamps and chemical etching (Ki et al., 2018), have also succeeded to imprint multilevel patterns in a single step. Large and complex eagle-shaped stamps (1 × 1 cm^2^, micrometer sized patterns) could be repeatedly imprinted on Si substrates, only limited in depth (< 1 μm) by mass transport of the electrolyte.

Another category of direct imprinting of silicon based on MACE concerns macroscopic 3D structures, i.e., of tens of micrometers either laterally or in height. The first example of silicon contact etching using a macroscopic object has been given in 2009 by the group of Matsumura (Lee et al., 2009). They used a platinum wire, 50 μm in diameter, anodically polarized against a counter-electrode in a HF solution and brought in contact with silicon to make cuts a few millimeters deep. Since then, grooves and through-holes have been etched using metal wires or tips as etching tools (Lee et al., 2009, 2011; Salem et al., 2010; Sugita et al., 2011, 2013). Another example of contact etching with a platinum needle has been given by Imamura et al. (2015). Takahashi et al. (2013) also reported on the use of platinum meshes (10 μm wires and 50 μm openings) attached to a sponge-like material to pattern arrays of macropores (10 μm in size).

The major problem encountered with electrochemical contact etching at the macroscopic scale is the diffusion of the electrolyte. Because the metal and silicon phases must be in intimate contact, there is no room for the diffusion of the electrolyte and consequently, as reported by Sugita et al. (2011), etching must proceed laterally from the solution bulk to the center of the metal tool, which slows down the in-depth etch rate. This problem is not severe when imprinting a single element with a diffusion path length of a few tenths of μm, (~wire diameter), but rather insurmountable for a “flat” contacting surface area of say a few cm^2^.

**Figure 1:**
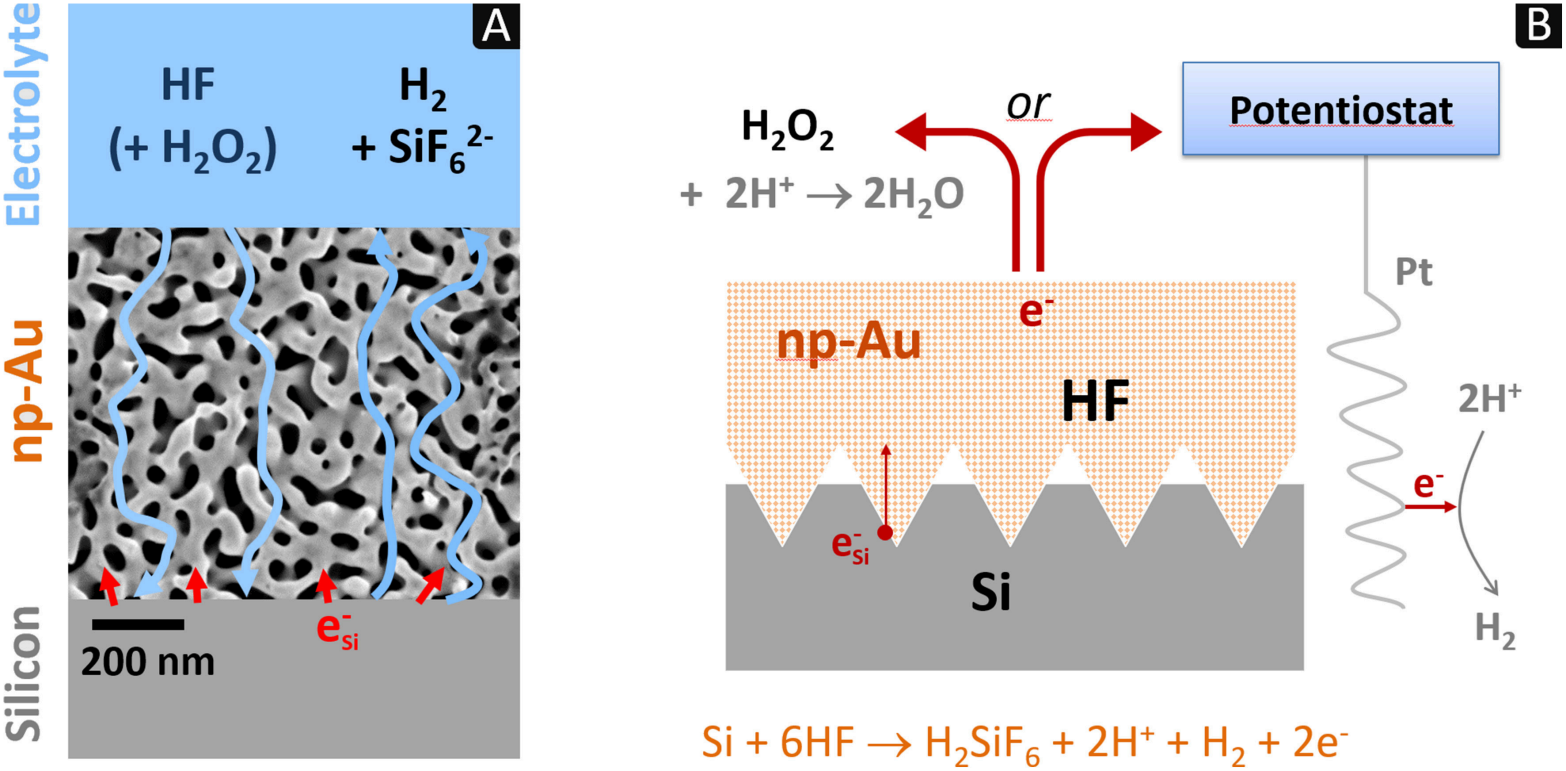
Scheme of the contact etching process: **(A)** diagram of the fluxes of electrons (red arrows) and chemicals (blue arrows) through a macroscopic piece of np-Au showing the nanometer-scale Au/Si contacts; **(B)** principle of 3D imprinting of silicon using a np-Au electrode with a pattern consisting in pyramids. Anodic polarization of the electrode can be provided either through an external circuit (electrochemical contact etching) or *in situ* by H_2_O_2_ (chemical contact etching).

We have recently reported a strategy specially defined to address this problem of large-scale electrolyte diffusion (Torralba et al., 2017). It is based on the use of nanoporous metal electrodes allowing both a “classical” MACE attack, i.e., with an interface consisting of nano-areas of metal and electrolyte in contact with silicon (for which the diffusion length is a few tens of nm) and a possible access of the electrolyte from the bulk of the solution to the interface. The principle of this configuration is represented in Figure 1A. The use of a nanoporous metal electrode to imprint patterns in silicon is a new concept. However, the well-known formation of silicon nanowires by MACE in HF-AgNO_3_ medium, as initially developed by Peng et al. (2002) is actually operating on the same principle. In this system, the oxidation of silicon atoms (dissolution) is coupled with the reduction of silver ions (deposition). During the experiment, silver grows dendritically from the silicon substrate to form a silver foam (*cf*. **Figure 7A** in Nassiopoulou et al., 2011) which reaches macroscopic dimensions (volume of ~1 cm^3^), much larger than the silicon substrate itself (*cf*. Materials and Methods and Figure E in **Supplementary Information**). This network of dendrites (~50 nm in diameter) is *de facto* a “nanoporous silver electrode.” The extremely localized dissolution of silicon at the Ag/Si interface leads to the formation of nanowires that lengthen as the “nanoporous silver electrode” sinks into silicon. The silicon nanowires are smooth and well-defined over several tens of micrometers, without parasitic etching. This is due to the depletion of silver ions inside the foam. They are only present outside of the foam (dendritic growth) but promotes the anodic polarization (Ag^+^/Ag redox couple) responsible for the electrochemical dissolution in HF of silicon at the Ag/Si interface. The anodic current corresponds to electrons injected (Si atom oxidation) at the bottom of the Ag foam and released at the tip of the dendrites (Ag deposition, *cf*. Figure E3 in **Supplementary Information**). This well-known experiment thus demonstrates the possibility of etching silicon on a macroscopic scale with a nanoporous metal tool (silver foam) allowing both electrolyte diffusion and MACE. The aim is therefore to develop an equivalent process with nanoporous metals of controlled structure and patterns.

Our first etching tests with nanoporous metal electrodes focused on imprinting square-based pyramids as represented in Figure 1B. They were made with nanoporous gold (np-Au) electrodes by electrochemical contact etching. A network of inverted pyramids was partially imprinted in n-type silicon oriented (100) in this way (Torralba et al., 2017).

We show this time that a complete transfer of complex patterns can be obtained in n-type silicon by contact etching, either electrochemically or chemically. The effects of several parameters such as the type of pattern (pyramid vs. U-shaped line), the characteristic size of the metal nanostructure (30 vs. 100 nm) and the nature of the catalytic metal (gold vs. platinum) with respect to etch rate, resolution of pattern transfer and formation of surface defects have been examined.

2D numerical simulations of the valence band modulation at the Au/Si/electrolyte interfaces have been performed to interpret the experimental results. The local modulations of silicon bands induced by metal nanocontacts were first studied by the group of Nakato (Nakato et al., 1988). This type of analysis subsequently benefited from numerical simulation methods and was reported by Rossi and Lewis for nanometer scale Ni arrays on Si electrodes (Rossi and Lewis, 2001) and by Huang et al. in the case of metal assisted electrochemical etching with silver nanoparticles on p-type silicon under anodic polarization in HF solutions (Huang et al., 2010). Recently, Torralba et al. studied MACE of p-type silicon with platinum nanoparticles using 2D numerical TCAD simulations based on a finite volume method (Torralba et al., 2016). The same type of modeling is developed here to determine the silicon band structure at adjacent metal and electrolytic contacts of nanometric size (20 nm). The assembly is connected so that metal contacts, which mimic the ligaments of the nanoporous metal electrode, can be anodically polarized with respect to the electrolyte through the silicon substrate (Metal/Silicon/Electrolyte). This allows to describe the band structure during etching.

## Materials and Methods

### Chemicals

AuAg alloys in the form of 170 nm thick leaves (12 carats, Au_35_Ag_65_, 8 × 8 cm^2^, Noris) were used as source of material for the elaboration of np-Au electrodes. Imprinting experiments were performed in n-type (phosphorous doped at N_d_~10^15^cm^−3^) c-Si wafers oriented (100), 1–3 Ω cm, 400 μm (Sil'tronix). Analytical grade (VWR chemicals) 30% H_2_O_2_, 96% H_2_SO_4_, 40% HF, 65% HNO_3_, 60% HClO_4_ and ultra-pure water (18.2 MΩ cm, Millipore) were used in all experiments.

### Electrochemical Treatments

Dealloying and contact etching were performed with a PGSTAT20 Metrohm Autolab and Nova software, in a three-electrode PTFE cell. The np-Au electrode, a platinum wire and a Hg/Hg_2_SO_4_ electrode (SME) were used as working, counter and reference electrodes, respectively. The SME included a K_2_SO_4_ bridge with a glass frit (EC dealloying) or a K_2_SO_4_ agar-gel tip (etching in HF).

### Instrumentation

Scanning electron microscopy (SEM) images, Energy Dispersive X-ray Spectroscopy (EDS) and Electron Back Scattered Diffraction (EBSD) were obtained with a Merlin FEG microscope from Zeiss equipped with AZtec systems (EDS Advanced, HKL Advanced Nordlys Nano, Oxford Instruments). Depth measurements of imprinted patterns were obtained by SEM from working distances measurements at different points of the patterns. AuAg powders were sintered using a Dr-Sinter 515S-Syntex Spark Plasma Sintering (SPS) machine.

### Fabrication of Silicon Molds

Alkaline etching using a SiN_x_ mask was used to create arrays of square-based inverted pyramids of 10 × 10 and 7 μm depth, in p-type (100) silicon wafers (5–10 Ωcm). Cryogenic plasma etching was used for parallel lines of rectangular cross section (U-shaped line) with a height of 5 μm, a width of 2, 5, or 10 μm and a spacing of 4 μm. In both cases, etching mask were defined by E-beam lithography.

### Patterned np-Au Electrodes

AuAg leaves were fragmented in water under simple magnetic stirring overnight and dried at 100°C. The obtained powder (~350 mg) was sintered against the silicon mold at 500°C under vacuum, with a uniaxial pressure of 50 MPa (3.3 kN/cm^2^) for 20 min, in a graphite die (Ø = 10 mm) enclosed between two graphite punches. After dissolution of the mold in HF-HNO_3_-H_2_O (44:16:40), Au_35_Ag_65_ disks of 10 mm diameter (0.35 mm thick) with a patterned central (79 mm^2^) were obtained. More details can be found in Supplementary Information.

### Electrochemical Contact Etching

n-type silicon wafers were cleaved into 2 × 2 mm^2^ pieces, cleaned in H_2_SO_4_-H_2_O_2_ (3:1) and rinsed with ultra-pure water. Before etching, the np-Au electrode was also cleaned in H_2_SO_4_-H_2_O_2_ (5:1). This must be performed with great care because the disproportionation of H_2_O_2_ into H_2_O and O_2_ is catalyzed by the gold surface and hence results in a strong effervescence of the hot mixture (exothermic mixing): the np-Au electrode was first placed in a Pyrex-type glass beaker with a small amount of H_2_SO_4_ and second H_2_O_2_ was added drop by drop. The electrode was cleaned for 5 min and then rinsed thoroughly with ultrapure water.

After this, the np-Au electrode was placed on a platinum plate (fixed on the bottom of a Teflon cell) which is itself connected to the potentiostat. A cleaned silicon piece was placed in contact with np-Au and a pressure applied *via* a weight in gold of 4.5 g. The electrolyte was 5 M HF with 2 vol.% ethanol to favor the elimination of H_2_ generated at the Si/metal interface.

### Chemical Contact Etching

The cleaning procedure of the sample and the electrode was the same as described above. The electrode was maintained under pressure (corresponding to a weight of ~0.4 g) using a gold wire connected to a micromanipulator. This is necessary due to H_2_ evolution (Si dissolution) and O_2_ evolution (disproportionation of H_2_O_2_ on Au) that may displace the electrode during imprinting. Etching was carried out in 5 mol L^−1^ HF and 1 mol L^−1^ H_2_O_2_, the rho value ([HF]/([HF]+[H_2_O_2_]) being 0.83.

### Use of Nanoporous Metal Electrodes

On the average, we could use 5 times np-Au electrodes with pyramids and 15 times those with U-shaped lines (for either chemical or electrochemical contact etching). With pyramids, “damaged tips” was the main reason for overruling an electrode. The second pattern is logically more robust, the damages originating more from handling. See Supplementary Information for more details.

Etch rate are most likely not constant during etching. We only have access to apparent etch rate, i.e., depth divided by etch time. For each set of etching condition, 3 to 4 experiments were used to calculate an average value of the apparent etch rates given in **Tables 2**, **3**. The maximum deviation from the average value is between 10 and 20%.

### Modeling

Numerical simulations in 2D of the valence band modulation at the Au/n-Si/electrolyte interfaces were performed using the commercial TCAD software [Atlas from Silvaco(Torralba et al., 2016)] based on a finite volume method. This simulator solves the physical equations governing the electrostatics (Poisson, electro-neutrality) and the transport of e^−^ and h^+^ (drift-diffusion) self-consistently on a 2D mesh.

The modeled structure is schemed in Figure D of **Supplementary Information**. It consists of a n-type silicon substrate with a thickness of 100 μm and a width of 1 μm, covered by 20 nm large gold pads, separated by two electrolyte contacts of the same length. The gold and electrolyte phases are separated by 1 nm of insulating vacuum to allow charge transfer only through the Si/Electrolyte and Si/Au interfaces. All the electrolyte contacts are short-circuited, so at the same potential, identically for the all gold pads. Silicon is doped n-type at a level of 3 × 10^15^ cm^−3^. The work functions of gold and the electrolyte are taken at W_Au_ = 5.5 eV (Hölzl and Schulte, 1979) and W_El_ = 4.5 eV (determined in our experimental conditions, *cf*. Torralba et al., 2016 and its Supplementary Information). The Fermi level is set at 0 eV at the equilibrium. To mimic the MACE process, a positive polarization can be applied between gold and the electrolyte.

## Results and Discussion

### Elaboration of Patterned np-Au Electrodes

#### Surface Patterns

The chosen strategy to elaborate patterned np-Au electrodes has been to design silicon molds using traditional microelectronics techniques and then sinter an Au_35_Ag_65_ powder in these molds to obtain their complementary shape (*cf*. experimental section). Experience has shown that the brittleness of silicon molds requires maintaining the pressure during sintering at the lowest possible value (3.3 kN) and that it was necessary to cover the silicon substrate with a Si_3_N_4_ diffusion barrier layer to avoid the formation of an AuSi eutectic which appears at 363°C. Under these conditions, a temperature of 500°C was chosen and the sintering time adjusted to obtain a good powder densification. The final density is at least 95% of that of bulk Au_35_Ag_65_ after 20 min of sintering.

**Figure 2:**
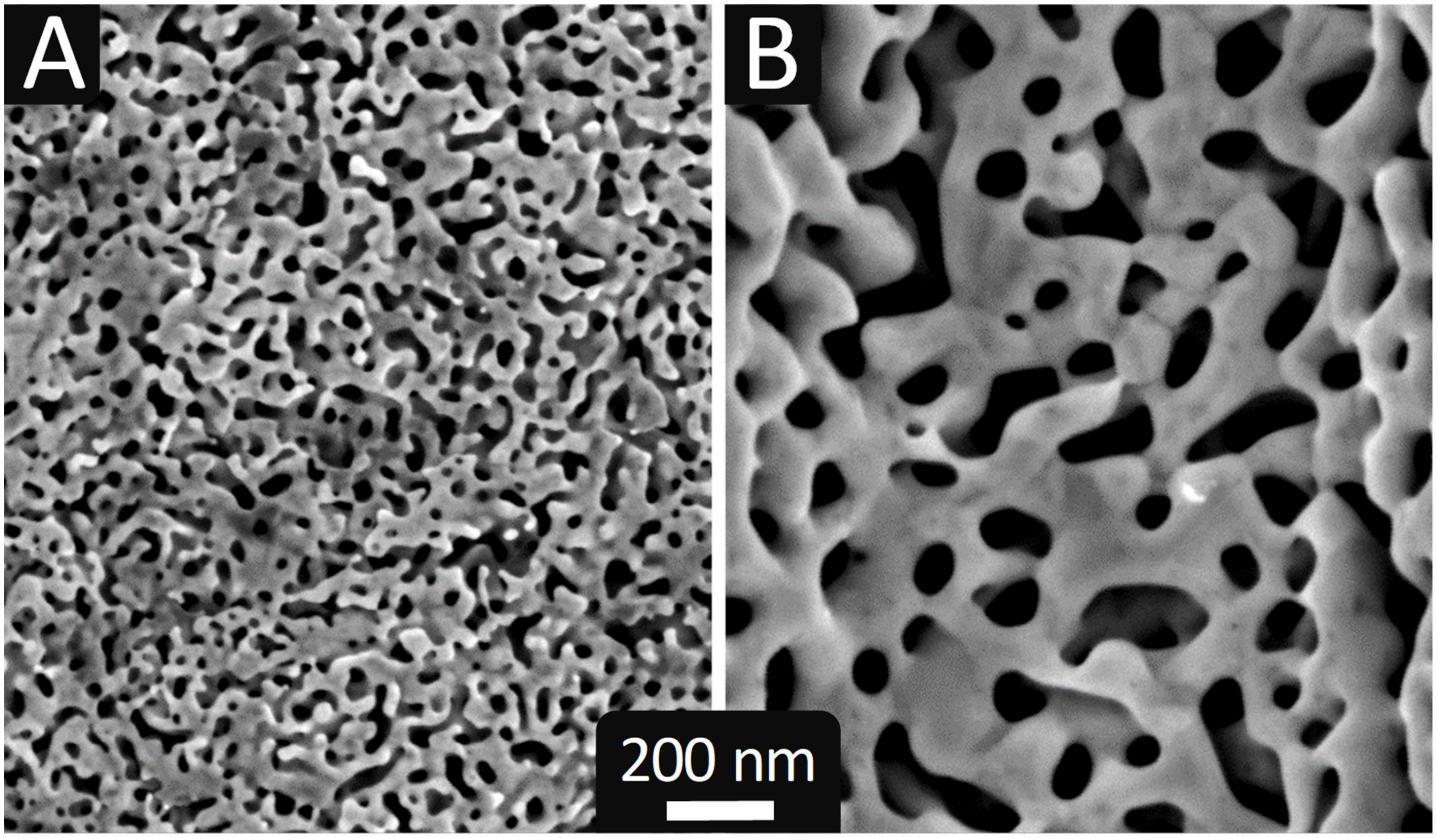
SEM images of np-Au electrodes elaborated by: **(A)** electrochemical dealloying at 0.7 V_SME_ in HClO_4_ (0.77 mol L^−1^) at 60°C for 53 h; **(B)** chemical dealloying in HNO_3_ (14.2 mol L^−1^, 65 wt.%) at 80°C for 12 h.

**Figure 3:**
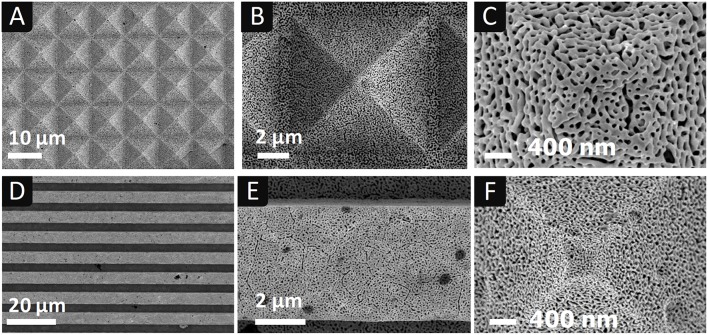
SEM images at different magnification of np-Au electrodes dealloyed chemically **(A–E)** and electrochemically **(F)**, with two different surface patterns: **(A–C,F)** array of square based pyramids (10 × 10 μm^2^ × 7 μm depth); **(D,E)** parallel lines with rectangular cross section (5 × 5 μm^2^ (width × height), spacing of 4 μm).

Au_35_Ag_65_ pellets, 300 μm thick, exhibiting arrays of square-based upright pyramids and U-shaped parallel lines on their surface were designed in this way. Figures 2, 3 show representative images of the patterned electrodes after being dealloyed as described below.

#### Au Nanostructures

Dealloying Au_35_Ag_65_, i.e., removing selectively silver, results in the formation of np-Au, a material consisting in a mixture of interconnected Au ligaments and pores which sizes can vary considerably (from 1 nm to 1 μm), depending on the dealloying conditions (Ding et al., 2004; Qian and Chen, 2007). The characteristic size of the nanostructure is a parameter that can influence the etching of patterns. Indeed, the dimensions of the ligaments determine the diffusion length that the electrolyte (reactants and dissolution products) must travel (parallel to the interface) to allow a complete dissolution of underlying silicon and thus the penetration rate of the electrode. The pore size may have an impact on the transport of the electrolyte but also on the escape of hydrogen bubbles formed by silicon dissolution in HF, when the dissolution valence *n* is < 4 (low anodic current, high HF concentration), according to the reaction:

$$\begin{array}{l} \text{Si}+6\text{HF}+{\text{nh}}^{+}\to {\text{H}}_{2}\text{Si}{\text{F}}_{6}+{\text{nH}}^{+}+\left[\left(4-\text{n}\right)/2\right]{\text{H}}_{2} \end{array}$$

We therefore wanted to develop nanoporous electrodes with different characteristic sizes to determine whether a fine or coarse nanostructure is more suitable for etching.

From the literature and trials, we have determined two sets of dealloying conditions to obtain nanostructures with very different sizes: electrochemical dealloying at 60°C in HClO_4_ at 0.7 V_SME_ for ~50 h and chemical dealloying in HNO_3_ at 80°C for 12 h. Etching times were established by weighing the AuAg pellet between successive treatments, i.e., by monitoring the removal of silver until zero mass loss is reached. Figure 2 shows SEM images of the pellets dealloyed with both treatments and Table 1 gives some features of the so formed np-Au.

**Table 1:** Characteristics of np-Au obtained by electrochemical and chemical dealloying.

**Dealloying mode**	**+ 0.7 V_**SME**_ in HClO_**4**_**	**HNO_**3**_**
Temperature, duration	60°C, 53 h	80°C, 12 h
Dissolved silver (wt.%)	87	100
Composition (EDS)—surface	Au_95_Ag_5_	Au
Composition (EDS)—core	Au_88_Ag_12_	Au
Average ligament width (nm)	28	95
Average pore area (nm^2^)	698	7,146
2D surface porosity (%)	22	28
Denomination	**fine np-Au**	**coarse np-Au**

Electrochemical dealloying produces a fine nanostructure (Figure 2A) whereas that obtained by chemical dealloying is coarse (Figure 2B). The average pore area varies over one order of magnitude and the average ligament width varies by a factor of 3 (Table 1). In agreement with these features, these two types of nanostructures will be designated hereafter as fine and coarse np-Au.

The porosity is difficult to evaluate. If we assume neither shrinkage nor expansion of the pellet during dealloying, the porosity of np-Au simply corresponds to the volume of dissolved Ag, which for Au_35_A**g**_65_ is 65% (silver and gold having nearly identical atomic radius). Accordingly, the porosity would be 59 and 65% for fine and coarse np-Au, respectively. The surface porosity, calculated by image analysis as the area fraction of pore openings (*cf*. details in Supplementary Information), is higher by a factor of ~1.3 for coarse np-Au vs. fine np-Au (28 vs. 22%, respectively).

Mass loss values indicate that silver removal is not completed after electrochemical dealloying. An EDS analysis conducted on the surface but also in the core of the pellet (after cleavage, at ~180 μm below the surface) confirms the residual presence of silver with a positive gradient toward the core. On the contrary, silver is not detected, even in the core, after chemical dealloying.

Figure 3 shows SEM images of np-Au electrodes with two different patterns, pyramids (A-D) and U-shaped lines (E-F), obtained by chemical (A-E) or electrochemical dealloying (F).

It can be seen that np-Au electrodes reproduce well the initial patterns of the silicon molds. The tip of the pyramids seems better defined when the characteristic size of np-Au is in the order of 30 nm, i.e., for fine np-Au, as could be expected. However, on all the electrodes produced, the replication resolution appears to depend firstly on the sintering step rather than on the type of dealloying. Indeed, several problems can occur at this stage which degrade the quality of the replica of the silicon mold patterns: areas contaminated by foreign particles/impurities deposited on the surface of the mold coming from the graphite die and punches, incomplete filling of the pyramid tips with the Au_36_Ag_65_ powder or even small breaks of the Si mold due to the applied pressure.

#### Metal Catalysts

In MACE of silicon, it is known that the nature of the metal catalyst has a profound effect on both the localization of dissolution around the catalyst and the dissolution rate. Platinum is more efficient than silver or gold at catalyzing the reduction of H_2_O_2_, which results in higher etch rate. However, it has been shown (with p-type silicon) that platinum generates a lot of porous silicon as a result of delocalized etching, while silver does not at all, and this can influence the resolution of pattern transfer. Hence, we wanted to perform tests with other nanoporous metals such as silver or platinum. These metals can be obtained in a nanoporous form but in a more difficult way and with a limited range in terms of characteristic size and porosity, thus making comparison with np-Au delicate. A simple and direct solution to this problem is to consider that, since catalysis of etching occurs at Si/metal contacts, it is sufficient to cover the np-Au surface with the desired metal. This allows to work under exactly the same conditions (ligament sizes, diffusion of the electrolyte into the electrode) but with another catalyst. As alternative catalysts, we tested Pt, deposited as 5 nm thick layers by sputtering on coarse np-Au electrodes. The SEM images of these electrodes are very similar to those of np-Au in Figures 2, 3 (not shown).

### Electrochemical Contact Etching

Etchings of silicon substrates were performed electrochemically in HF by anodic polarization of np-Au electrodes. Table 2 summarizes the etching conditions tested and the corresponding results. The highlights that can be drawn from these results are presented in the following sections through SEM images of the imprinted surfaces.

**Table 2:** Etching conditions and results obtained by electrochemical contact etching using np-Au electrodes with pyramids and U-shaped lines arrays as surface patterns (*cf*. Supplementary Information for more details on contact etching data).

**Metal**	***Pattern***	**Au nanostructure *Remaining Ag* < ligament size>**	**Electrochemical contact etching in HF (5 mol L^**−1**^ HF + 2 vol.% EtOH)/apparent etch rate (depth/etch time)**
np-Au	*Pyramid*	fine^a^ *12 wt.%* < 28 nm >	0.3 V_SME_, 10 min → partial imprinting/0.36 μm min^−1^
	__________		_______________________
	*Line*		0.3 V_SME_, 20 min → 0.3 μm deep/0.015 μm min^−1^ *PSi ^*c*^ traces + Ag deposition*
np-Au	*Pyramid*	coarse^b^ *0 wt.%* < 95 nm >	0.2 V_SME_, 20 min → full imprint/0.35 μm min^−1^
	__________		_______________________
	*line*		0.2 V_SME_, 20 min → 1.5 μm deep/0.075 μm min^−1^

a 0.7 V_SME_, 53 h;

b HNO_3_, 80°C, 12 h;

c *porous silicon*.

#### Influence of the Nanostructure Size

**Figure 4:**
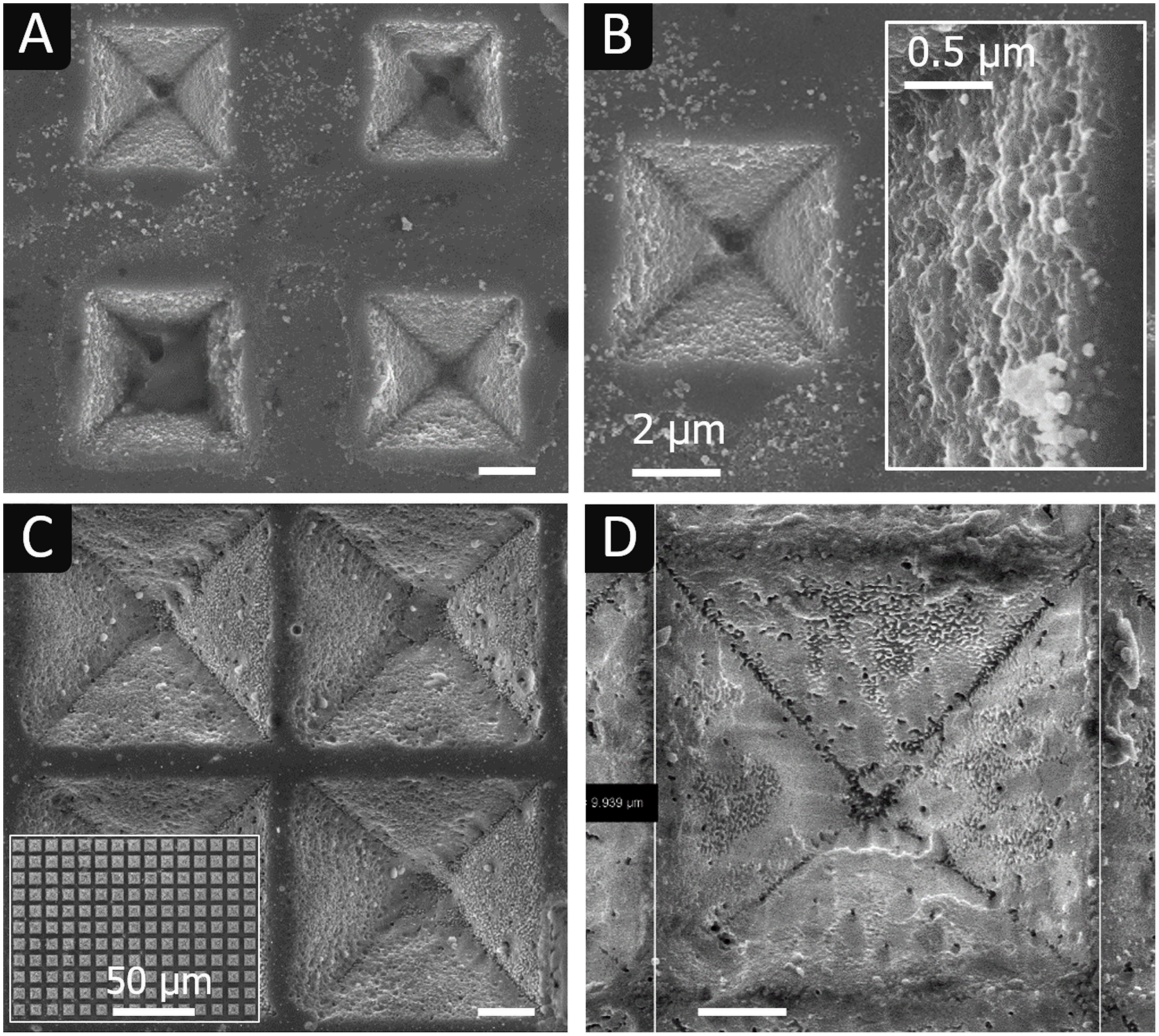
SEM images of silicon surfaces after imprinting inverted pyramids with a fine **(A,B)** and a coarse **(C,D)** np-Au electrode. Electrochemical contact etching was carried at 0.3 V_SME_ for 10 min **(A,B)**; at 0.3 V_SME_ for 20 min **(C)** and at 0.2 V_SME_ for 20 min **(D)**. Insets: **(B)** inverted pyramid border at higher magnification; **(C)** inverted pyramid array at lower magnification. Electrolyte: HF 5 mol L^−1^ with 2 vol.% EtOH. Scale bars: 2 μm unless otherwise noted.

Figure 4 shows examples of imprinted silicon surfaces with fine and coarse np-Au pyramids. With fine np-Au electrodes, inverted pyramids of 5 × 5 μm^2^ (3.6 μm depth) were etched after 10 min at 0.3 V_SME_ (Figures 4A,B). With coarse np-Au electrodes, inverted pyramids of 9 × 9 μm^2^ were patterned after 20 min at 0.**3** V_SME_ (Figure 4C). In another experiment, conducted at 0.2 V_SME_ for 20 min with the same electrode, full pattern transfer was achieved (Figure 4D): the inverted pyramid base is 9.9 μm and the depth 7 μm at the tip. It results in a lack of flat spots between adjacent inverted pyramids. The maximum apparent etch rates (depth/etch time), after several experiments with both types of electrodes) are found to be relatively close (0.32–0.36 μm/min). Hence, the characteristic size of the gold nanostructure does not affect the apparent etch rate significantly.

A comparison of Figures 4B,D shows that the surface state is different depending on the type of np-Au (fine or coarse). The insert in Figure 4B is a zoom on an edge of the pyramid. The surface appears rough and there is silver deposited in the form of nanoparticles (as indicated by EDS). This deposit, also visible on the flat part of the substrate between pyramids (Figure 4A) must be related to the residual presence of silver in the electrodes (5–12 at.%, *cf*. Table 1). During etching, the anodic polarization of the electrode causes some dissolution of silver and the silver ions thus formed diffuse to the silicon substrate. Their reduction into silver nanoparticles is coupled with silicon dissolution, which causes craters to form around the nanoparticles and is thus at the origin of the surface roughness (Chemla et al., 2003; Chartier et al., 2008; Lee et al., 2008).

Inverted pyramids patterned with coarse np-Au (Figure 4D) have both smooth and rough areas, with some porous silicon visible on the upper edges. The absence of silver deposition is consistent with the complete dealloying obtained chemically in HNO_3_ at 80°C (*cf*. Table 1). It is interesting to note that a complete pattern transfer of the pyramid network is achieved after 20 min etching at 0.2 V_SME_ (Figure 4D) and quasi-complete at 0.3V_SME_ (same electrode and etching time) (Figure 4C). Below 0.2 V_SME_, imprinting was found to be much less effective (only the pyramid tips were visible on the silicon surface) due to a low oxidation current. The bias applied to etch at 0.2 V_SME_ was ~ 0.35 V (V_OCP_ ~ −0.15 V_SME_).

#### Effect of the Pattern

**Figure 5:**
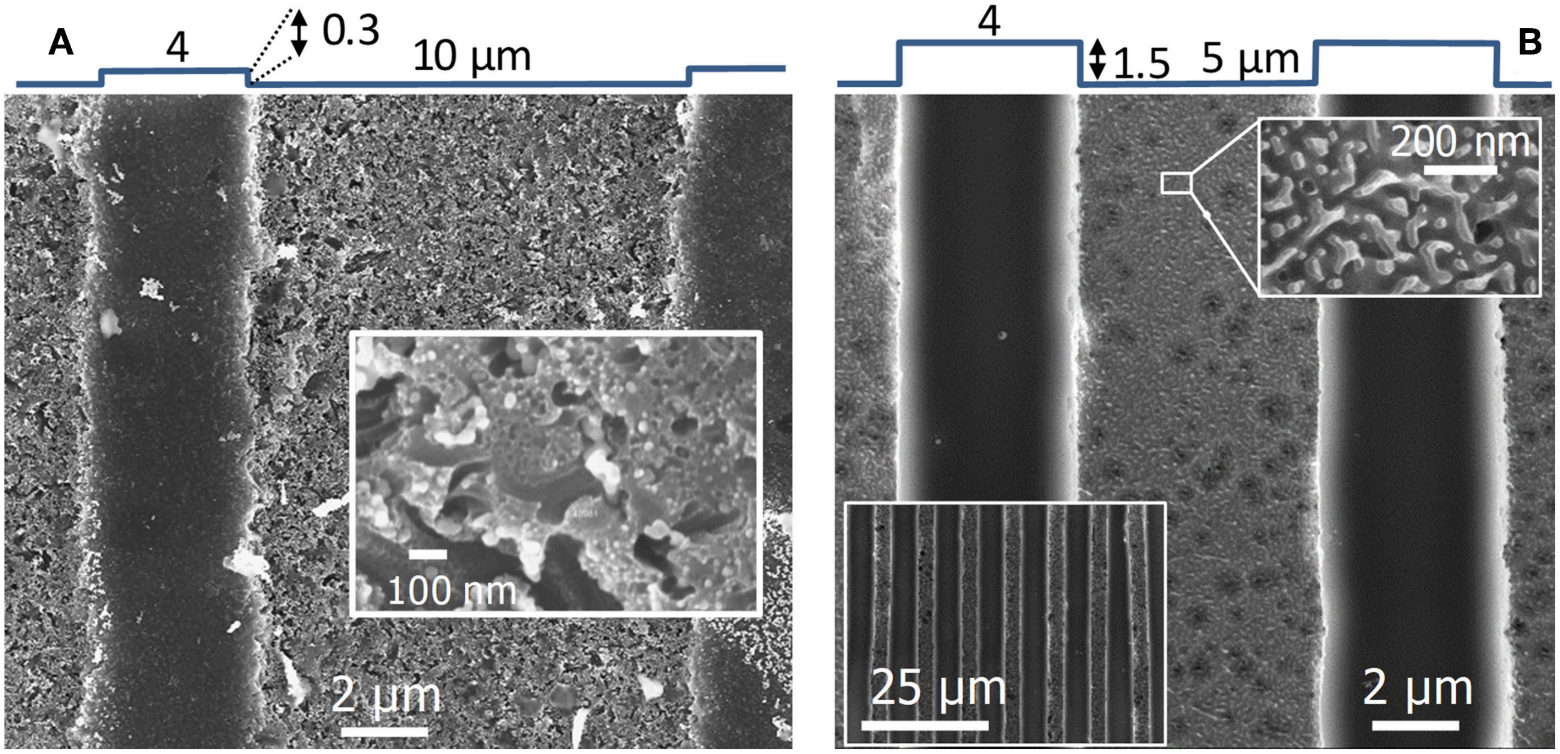
SEM images of silicon surfaces after imprinting U-shaped lines with np-Au electrode: **(A)** fine nanostructure (0.3 V_SME_, 20 min*)*; **(B)** coarse nanostructure (0.2 V_SME_, 20 min). Insets: **(A, B-upper)** high magnification image of the etched area; **(B-bottom)** U-shaped line array at a lower magnification. Electrolyte: HF 5 mol L^−1^ with 2 vol.% EtOH.

An example of trenches patterned in silicon by electrochemical contact etching of np-Au electrodes with U-shaped lines is shown in Figure 5.

Almost no imprinting (0.3 μm in depth) was obtained with np-Au electrodes of fine nanostructure, as shown in Figure 5A. The surface is extremely damaged as a result of silver deposition, as clearly shown in the inset image at higher magnification of the etched surface. Deeper imprints were observed when using electrodes with coarse np-Au, and without silver deposition (Figure 5B), in agreement with the results obtained with pyramids. The etched bottom of the trenches exhibits the details of the electrode coarse nanostructure (upper inset of Figure 5B), the small silicon walls corresponding to pore openings. This indicates a high pattern transfer resolution. From these results, coarse np-Au seems to be more suitable than fine np-Au for imprinting.

In terms of imprinting depth, etching was more effective for the pyramid pattern (*cf*. Figures 4, 5). The maximum depth reached with U-shaped lines under identical etching conditions (0.2 or 0.3 V, 20 min) was 1.8 μm compared to 7 μm for pyramids. The penetration rate is therefore almost four times lower for the U-shaped line pattern. This difference could be related to the supply of electrolyte at the interface. However, the diffusion path of the electrolyte through a 300 μm thick nanoporous electrode is essentially the same regardless of the surface pattern (~10 μm in height). It is possible that the actual difference lies in the free volume of electrolyte around the patterns which is more confined in the case of U-shaped lines (flow only along the lines) than for the pyramids thanks to their truncated shape. This implies that despite the porous nature of the electrode there could be some limitation in electrolyte supply.

### Chemical Contact Etching

Admittedly, the use of np-Au electrodes allows to apply a polarization externally and this offers a reproducible control of the potential, the ability to measure the dissolution current and avoids the use of an oxidizing agent. However, because in some cases it is simpler to use the purely chemical method of classical MACE, etching tests were conducted in HF-H_2_O_2_ solutions, as reported in Table 3.

**Table 3:** Etching conditions and results obtained by chemical contact etching using np-Au and np-Au/Pt electrodes with pyramid and U-shaped line arrays as surface patterns (*cf*. Supplementary Information for more details on contact etching data).

**Metal**	***Pattern***	**Au nanostructure *Remaining Ag* <ligament size>**	**Chemical contact etching in HF/H_**2**_O_**2**_ (5:1 mol L^**−1**^)/apparent etch rate (depth/etch time)**
np-Au	*Pyramid*	fine *12 wt.%* < 28 nm>	20 min → partial imprinting/0.28 μm min^−1^
	__________		_______________________
	*Line*		20 min → no imprinting
np-Au	*Pyramid*	coarse *0 wt.%* <95 nm >	20 min → full imprint/0.35 μm min^−1^
	__________		_______________________
	*Line*		20 min → 2.8 μm deep/0.14 μm min^−1^
____________			_______________________
np-Au/Pt^*a*^	*Pyramid*		25 min → full imprint/0.28 μm min^−1^
	__________		_______________________
	*Line*		25 min → 1.7 μm deep/0.07 μm min^−1^

a *5 nm coating on np-Au*.

Compared to electrochemical etching, we found that a higher gas evolution occurs in HF-H_2_O_2_. It is most probably related to the disproportionation of H_2_O_2_ on the gold surface of the electrode.

**Figure 6:**
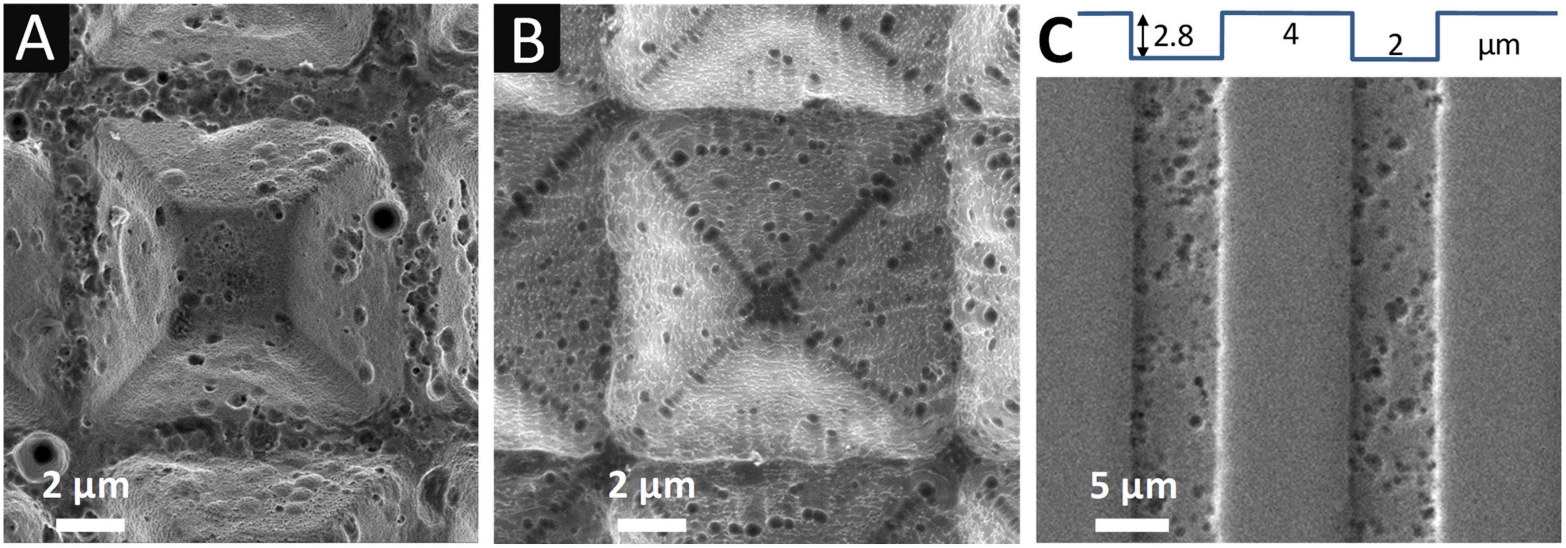
SEM images of silicon surfaces after imprinting inverted pyramids in HF-H_2_O_2_ with **(A)** fine np-Au and **(B)** coarse np-Au, and U-shaped lines with **(C)** coarse np-Au/Pt electrodes. Electrolyte: HF 5 mol L^−1^ - H_2_O_2_ 1 mol L^−1^, with 2 vol.% EtOH.

As for electrochemical contact etching, while fine np-Au electrodes did not provide a full pattern transfer (Figure 6A), a complete transfer was obtained with coarse np-Au electrodes (Figure 6B). Inverted pyramids have sides of 10 μm and a depth of 7 μm, like their models. The surface is relatively rough and craters of ~ 500 nm in diameter and ~ 100 nm deep are present everywhere. The intersections of facets are relatively rounded, indicating a partial delocalization of etching, probably due to porous silicon formation. SEM observations in cross section confirm the rounded edge morphology and the presence of a porous silicon layer everywhere on the walls, with a thickness of ~50 nm measured at the bottom of an inverted pyramids (*cf*. Figure G in **Supplementary Information**). These defects were not observed after electrochemical contact etching. Delocalized etching with porous silicon formation is a well-known effect of MACE that explains the production of tapered and porous silicon nanowires with metal mesh as catalyst [(Azeredo et al., 2013) and (Geyer et al., 2012), respectively] or that of cone-shape pores with platinum nanoparticles (Torralba et al., 2016). However, delocalized etching is not expected with low doped n-type silicon and gold or platinum catalysts. This discrepancy is discussed in the Modeling section.

Trenches imprinted chemically with coarse np-Au (Figure 6C) exhibit a depth of 2.8 μm which is two times higher than what is obtained electrochemically (1.5 μm). Like with the inverted pyramids (Figure 6B) the etched surface (bottom) presents a high density of craters.

#### Effect of the Metal Catalyst

**Figure 7:**
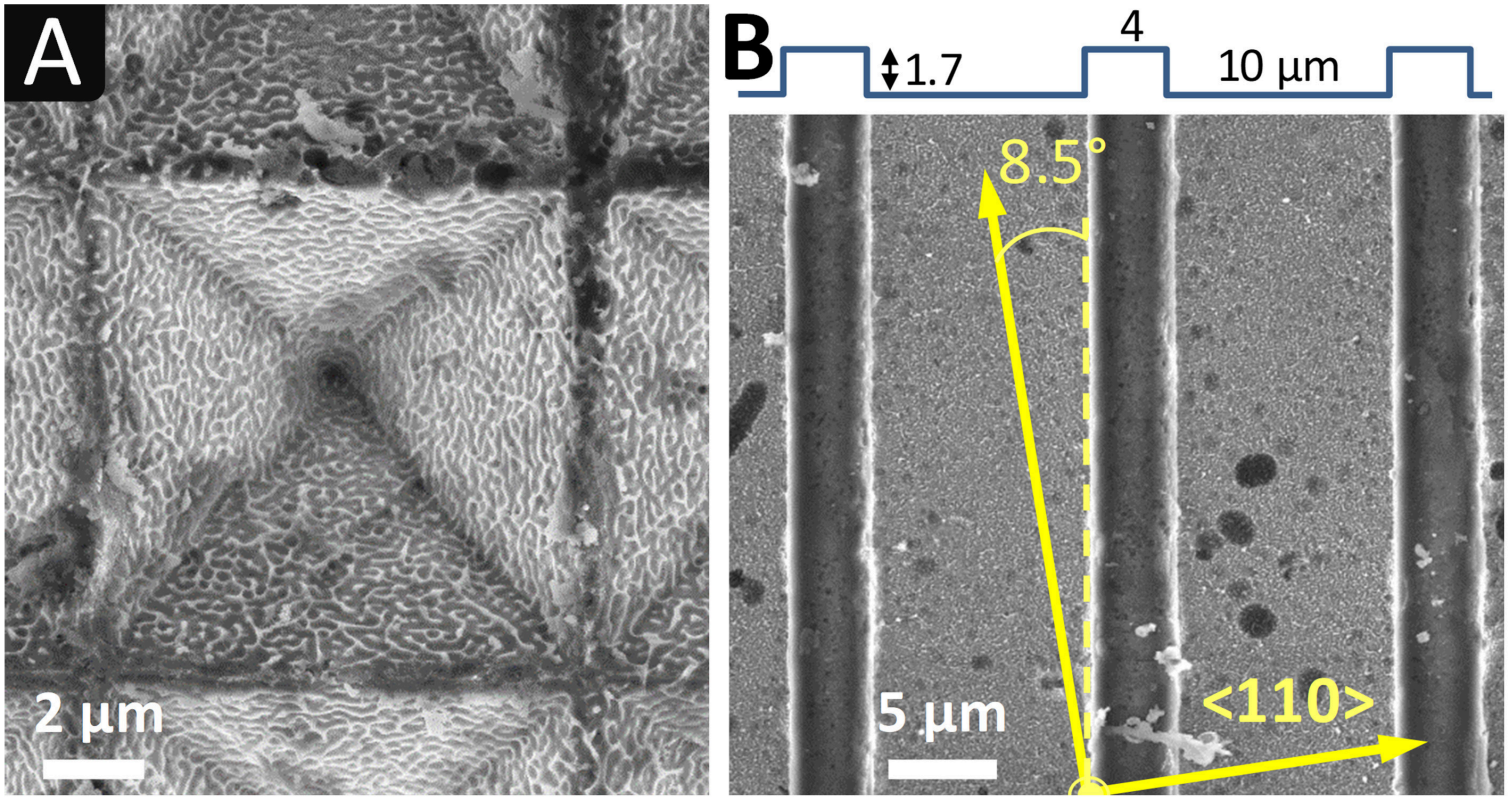
SEM images of silicon surfaces after imprinting chemically **(A)** inverted pyramids and **(B)** U-shaped lines in HF-H_2_O_2_ with coarse np-Au/Pt electrodes. Electrolyte: HF 5 mol L^−1^ - H_2_O_2_ 1 mol L^−1^, with 2 vol.% EtOH. In **(B)**: line offset with respect to < 110> directions, as determined by EBSD.

In order to study the influence of the metal catalyst, inverted pyramids and trenches were imprinted chemically with np-Au/Pt electrodes, under the conditions reported in Table 3. SEM images of the obtained inverted pyramids and trenches are given in Figures 7A,B, respectively.

The chemical apparent etch rate with coarse np-Au/Pt is lower than that obtained with coarse np-Au, the trench depth after 20 min being 1.7 μm and 2.8 μm, respectively. It should be noted though that the U-shaped lines with np-Au/Pt are five times larger than in the case of np-Au (10 μm instead of 2 μm, identical spacing of 4 μm). The volume of etched silicon is therefore five times more important, which is consistent with the difference in apparent etch rates.

Another difference lies in the quality of the pattern transfer, which seems slightly better with Pt: the facets of pyramids are flatter and their intersections better marked; very few craters are present although a significant amount of porous silicon appears in the form of particles detached from the surface. The surface state is also different, with the presence of “small walls” that appear to be a replica of the pores on the surface of the metal electrode. Such walls are also observable at some locations on the surface of silicon electrochemically etched by anodization with a np-Au electrode (see Figures 4B, 5B). Hence, their presence is not due to the intrinsic properties of gold and platinum (work functions), or to the contact etching mode, but to certain etching conditions that leads to a higher transfer resolution.

Figure 7B also shows that the trenches are not aligned with the < 110> directions of the substrate because the electrode and silicon samples are contacted regardless of their orientation. These directions are those necessarily followed in the case of alkaline etching and lithography (Moreau, 1988). In the same vein, it should be stressed that the facets of the pyramids obtained by contact etching do not correspond to (111) planes as is the case with alkaline etching (Torralba et al., 2017). This independence from the crystallographic orientation is obviously an advantage for creating new or more complex patterns.

### Modeling

**Figure 8:**
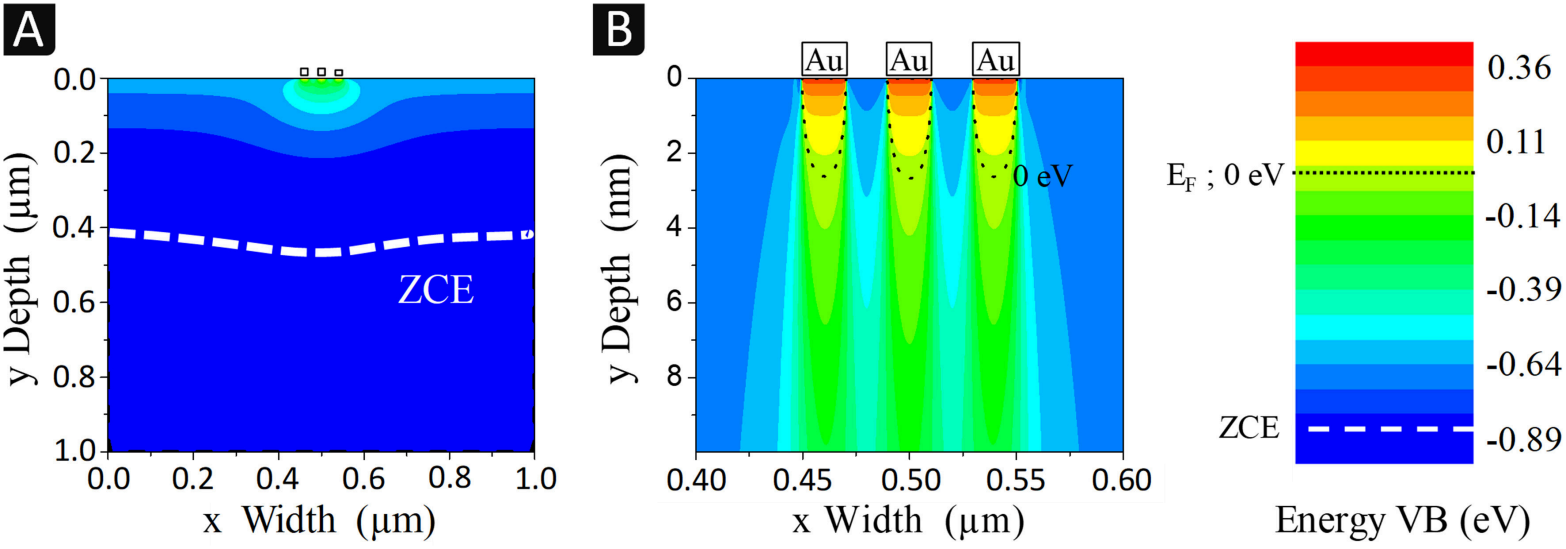
Simulation of 3 gold ligaments (20 nm in size and interspace) surrounded by an electrolyte and in contact with n-type silicon (3 × 10^15^ cm^−3^) through 2D profiles of the valence band energies (referenced to the Fermi level) at equilibrium, at low **(A)** and high **(B)** magnification. The VB energy color scale is given on the right-hand side.

To explain the results obtained experimentally, numerical simulations of the modulation of the silicon valence band induced by the metal (ligaments) and the electrolyte contacts (pores) were performed at the scale of a few tenths of nanometers. For that, we developed a 2D model representing a Si/Electrolyte interface over a distance of 1 μm with 3 gold pads at the center, 20 nm in size and interspacing and surrounded with electrolyte, as described in the experimental section (*cf*. Figure D in **Supplementary Information**). The size of the pads was chosen small enough to apply to both fine (30 nm) and coarse (100 nm) Au nanostructures. Modulations obtained with 20 nm plots and interspacing will only be accentuated at larger sizes. Figure 8 shows the 2D variations of the valence band laterally and in depth (at low (A) and high magnification (B)) at equilibrium (0 V).

Figure 8A reveals that the silicon valence band is modulated by the gold contacts only at close distance to the surface (< 0.1 μm). Figure 8B shows that most of this modulation follows the details of the “np-Au nanostructure” whose ligaments are represented by the gold pads.

**Figure 9:**
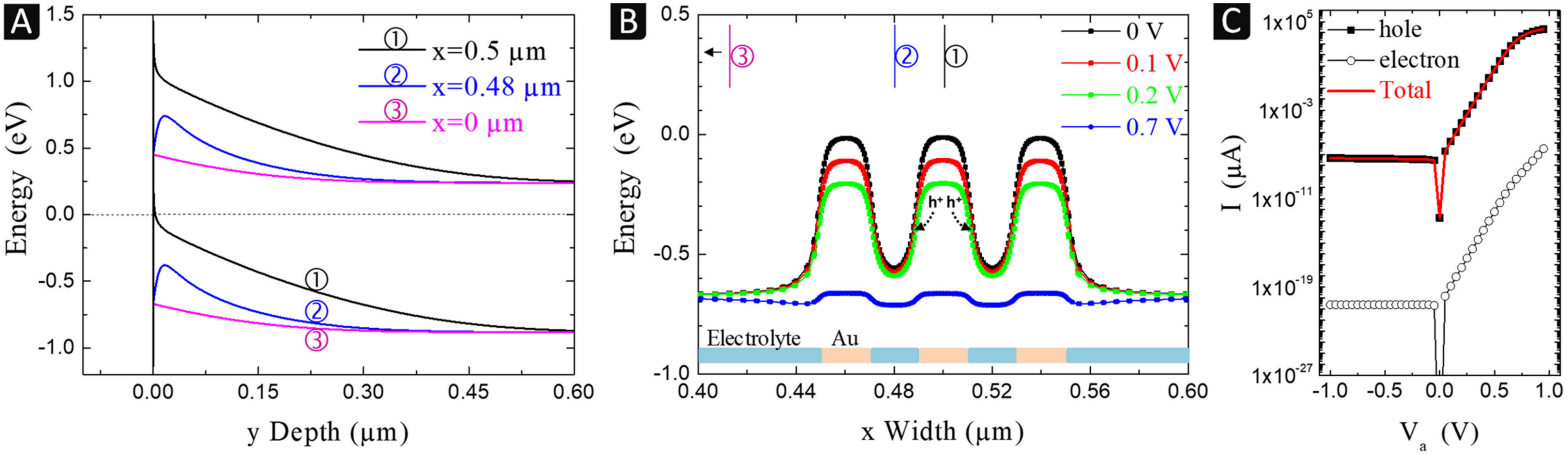
Simulations of: **(A)** the band bending of n-Si/Au and n-Si/electrolyte junctions with cut-lines (y Depth) corresponding to a gold pad center (x = 0.5 μm), an electrolyte contact between two gold pads (x = 0.48 μm) and the electrolyte far from gold (x = 0 μm); **(B)** lateral modulations (x Width) of the valence band energies at 3 nm beneath the silicon surface, at 0 V (equilibrium) and under 0.1, 0.2, and 0.7 V bias applied to the gold contacts (*cf*. Supplementary Information); **(C)** *I-V* characteristics of the Au/n-Si/Electrolyte device.

Figure 9A gives the 1D band diagrams for three cut-lines, one centered under a gold pad (x = 0.5 μm), and the other under an electrolyte contact either between two gold pads (x = 0.48 μm) or far from them (x = 0 μm). From this figure, it can be seen that the two phases (gold and electrolyte) form nano-Schottky junctions of different barrier heights, with an intermediary situation between gold pads.

At the level of Au, the valence band exceeds the Fermi level, which results in a strong inversion regime with an accumulation of holes (valence band above E_F_) within the first 3 nm, equivalent to a 2D hole gas. To simulate the electrochemical or chemical etching process, an anodic bias is applied to the np-Au pads with respect to the electrolyte. Figure 9B shows the lateral modulations of the valence band energy at the border of the hole accumulation region (3 nm underneath the silicon surface) under positive polarization. The anodic bias of gold (0.1, 0.2 V, and 0.7 V) results in a potential drop occurring mostly at the n-Si/Au interface and slightly at the n-Si/electrolyte junction, especially at the edge of the gold pad (the potential barrier of the Si/Electrolyte junction remains nearly the same). At low applied bias, holes located under the gold pads face an energy barrier and can barely diffuse laterally whereas at high polarization (>0.7 V), this barrier almost disappears which induces a lateral hole flow. The *I-V* curves corresponding to the current flowing through the device (two interfaces in series, Au/n-Si/electrolyte) are reported in Figure 9C. These simulated curves makes it possible to determine that the etching current is due to a flow of holes despite the n-type doping of Si (holes being minority carriers in the bulk and majority carriers in the inversion layer at the surface).

The simulation results show that the current remains laterally confined very close to the surface, without exchange with the bulk, i.e., without delocalized etching next to the electrode or on the back side of the silicon sample. This agrees with the experimental results described previously. This effect, highly desired because it favors a localized etching around the gold ligaments, is mainly due to the n-type doping which does not lead to an ohmic but rectifying contact with Au, i.e., to a potential barrier that prevents holes from diffusing toward the bulk. Since W_Pt_ and W_Au_ are almost identical (5.7 vs. 5.5 eV, respectively), from a band bending and carrier transport point of view, the situation would be very similar with Pt, in agreement with the ability to imprint pyramids observed with both metal catalysts. On the contrary, in the case of a p-type silicon, the contact is ohmic with both gold and platinum, and therefore there is a delocalized etching, as described for the Pt/p-Si system (Torralba et al., 2016). The silicon walls observed in Figure 7A, for example, result from this localization of the etching under the ligaments and not (or less) between them (pores). The height of the walls is nevertheless limited by deeper ligaments in the electrode that come into contact with them as the electrode penetrates the substrate. Under higher positive bias (0.7 V), the lateral band bending at the interface between Au and electrolyte contacts can be canceled (Figure 9B) and the diffusion of holes away from n-Si/Au interfaces is possible. The presence of porous silicon observed after chemical etching may be related to this phenomenon. The high oxidative power and concentration of H_2_O_2_ combined with the large surface area of np-Au can result in a significantly reduced diffusion barrier and a high current of holes along the inversion layer, causing porous silicon to form in areas between or away from the metal ligaments. A more precise study of this effect is needed in future work.

## Conclusion

The process of patterning silicon at the macroscopic scale by contact etching with nanoporous metal electrodes has been developed in several directions. We have shown that a complete transfer of complex patterns such as square-based pyramids can be obtained by electrochemical or chemical contact etching in n-type Si. The apparent etch rate depends on the type of imprinted patterns, being two to four times lower for U-shaped lines than for pyramids. Using np-Au electrodes of different characteristic sizes (30 and 100 nm) has revealed that this parameter has little influence on pattern transfer. Finally, the influence of the metal catalyzing the etching could be tested under identical conditions by covering the surface of a np-Au electrode with a platinum layer (5 nm). “np-Pt” is also suitable to transfer the pyramid pattern and seems to offer a better resolution.

The surface state after imprinting presents defects (roughness, pores, craters, porous silicon). When np-Au with a fine structure is used, the presence of residual silver in the electrode (related to the dealloying method used to produce this fine structure) strongly degrades the surface state due to silver deposition during etching (formation of craters or pores). With np-Au of coarse structure, the resolution is better, with even the formation of small walls due to imprinting of the gold nanostructure details (ligaments and pores). By simulating the modulation of the valence band on a scale of a few tens of nanometers, we have shown that there are holes under the metal (within 3 nm) because of the inversion regime established with n-type silicon in contact with gold or platinum. A lateral potential barrier in silicon between regions in contact with the metal and the electrolyte prevents these holes to diffuse. Under moderate anodic polarization, etching corresponds to a quasi 2D electron transfer from a metallic contact zone to the adjacent electrolytic zone and is therefore very localized, which explains the formation of small walls at the level of pores in np-Au(/Pt). If the anodic polarization (electrochemical or chemical) is higher, the band bending between the contacts and the electrolyte can be canceled and the dissolution becomes more delocalized without formation of walls.

All these results demonstrate the interest of imprinting 3D patterns in silicon using nanoporous metal electrodes. Further investigations are needed to increase the etch rate, improve the surface quality and the tolerance to electrode's defects and to test new and more complex pattern transfers.

## Author Contributions

All authors participated in the design and planning of the research. MH, VM, JH, DY, and J-PV worked on the elaboration of Si molds. ET, EM, and SB performed the patterning experiments and SL the numerical simulations. SB and ET wrote the first draft of the manuscript. All authors provided feedback and participated to the final writing of the manuscript.

### Conflict of Interest Statement

The authors declare that the research was conducted in the absence of any commercial or financial relationships that could be construed as a potential conflict of interest.
