# Supplementary material for: 3D Patterning of Si by Contact Etching With Nanoporous Metals
Source: Front Chem. 2019 Apr 25;7:256. doi: 10.3389/fchem.2019.00256 (PMC6494945; doi:10.3389/fchem.2019.00256)
Supplement: Supplementary file 1 [file Data_Sheet_1.PDF]

3D patterning of Si by contact etching with nanoporous metals

Supplementary Information

Stéphane Bastide[1*], Encarnacion Torralba[1], Mathieu Halbwax[2], Sylvain Le Gall[3], Elias Mpogui[1], Christine Cachet-Vivier[1], Vincent Magnin[2], Joseph Harari[2], Dmitri Yarekha[2], Jean-Pierre Vilcot[2]

[1] Université Paris Est, ICMPE (UMR 7182), CMTR, CNRS, UPEC, F- 94320 Thiais, France

[2] Institut d'Électronique, de Microélectronique et de Nanotechnologie, CNRS, Université Lille-1, F-59 652, Villeneuve d'Ascq, France

[3] Génie Électrique et Électronique de Paris (GeePs), CNRS, Centrale-Supelec, Université Paris-Sud, Sorbonne Université, F-91192 Gif sur Yvette, France

*** Correspondence:** Stéphane Bastide. bastide@icmpe.cnrs.fr

1. Materials and Methods

Patterned np-Au electrodes. AuAg leaves were fragmented in water under simple magnetic stirring overnight and dried at 100°C. The obtained powder (~350 mg) was sintered against the silicon mold at 500°C under vacuum, with a uniaxial pressure of 50 MPa (3.3 kN/cm^2) for 20 min, in a graphite die (Ø = 10 mm) enclosed between two graphite punches. After dissolution of the mold in HF-HNO_3-H_2O (44:16:40), $Au_{35}Ag_{65}$ disks of 10 mm diameter (0.35 mm thick) with a patterned central (79 mm^2) were obtained. More details can be found in (Torralba et al., 2017). Figure 1 shows the SPS instrument used.

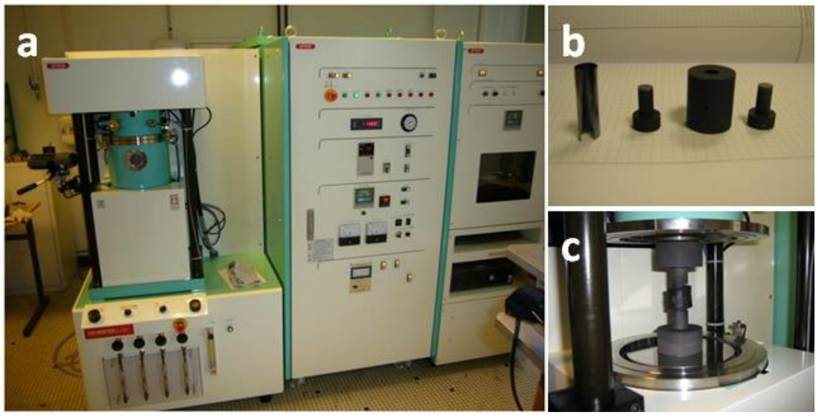

Figure A. Spar Plasma Sintering (SPS with photos of (a) the whole instrument, (b) the graphite die and punches before and (c) after installation in the SPS instrument.

Elaboration of AuAg powders. For the synthesis of the nanoporous electrodes, we use a bimetallic alloy of $Au_{35}Ag_{65}$. These are 12-carat white gold leaves made by Noris Blattgoldfabrik **(***cf.* Figure B**)**. They have a composition of [50:50] %.m, [35:65] at.%, and a thickness of 170 nm (which makes their handling delicate**).**

The AuAg leaves are fragmentated by sonication. They are immersed in ultra-pure water (UP) and fragmented at a power of 55 W for 10 min. The resulting solution is then dried in an oven at 100°C for 8 hours. Alternatively, fragmentation can also be done under simple magnetic stirring. The leaves are placed in an aqueous solution and stirred with a magnetic rod for 8 hours and then dried in an autoclave at 100°C. **Figure B** details the evolution of the AuAg leaves fragmentation during this process and shows a SEM images of the obtained fragments.

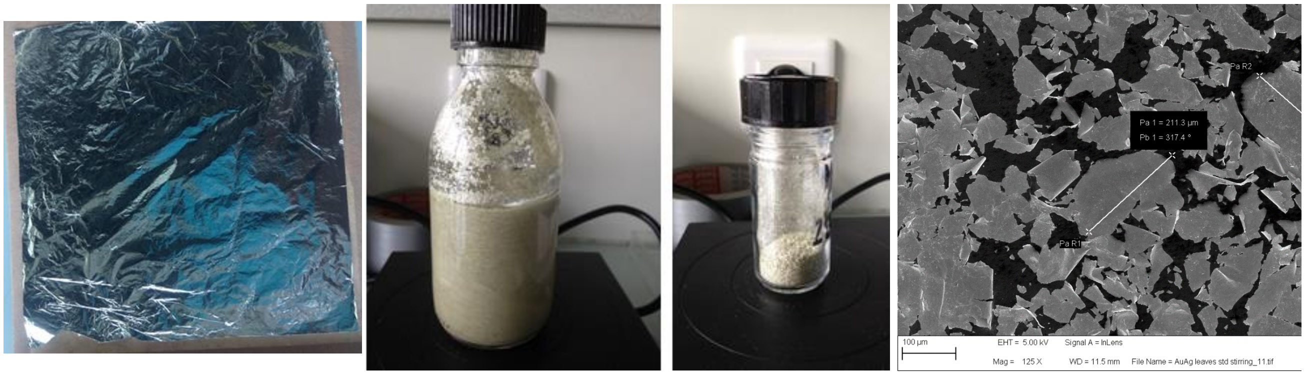

Figure B : Photos, from the left: a white gold leaf ($Au_{35}Ag_{65}$); fragmentation under magnetic stirring in UP water; powder eventually obtained (after 8 h). SEM image: $Au_{35}Ag_{65}$ fragments.

The resulting powder is sintered by SPS in Si molds with different patterns to obtain an $Au_{35}Ag_{65}$ electrode. To do this, the powder (~350 mg) is placed in the graphite die, with the side containing the patterns in contact with the powder. Figure C-Left shows a SEM image with some metal powder fragments on a silicon substrate with pyramids (obtained by alkaline etching without lithography) of the same size than the inverted pyramids (obtained by alkaline etching with lithography) employed in the study.

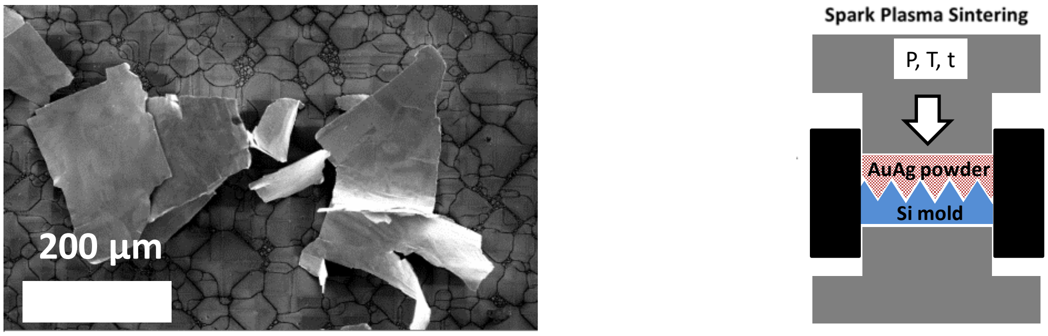

Figure C. (Left) $Au_{35}Ag_{65}$ fragments (obtained by sonication) deposited on a silicon substrate with random pyramids obtained by alkaline etching (without lithography); (Right) Scheme of the sintering experiment geometry.

Under the effect of pressure and temperature, the powder fills the patterns and is sintered into a solid piece, as shown in the scheme of Figure C-Right. We have established that the optimal data for the

This is a provisional file, not the final typeset article

sintering were: an increase in temperature from 20 to 500°C in 5 min; a plateau at 500°C for 20 min; a temperature drop from 500 to 20°C in 45 min; a constant pressure of 3.3 kN (50 MPa).

Modeling. The modeled structure is schemed in Figure D. It consists of a n-type silicon substrate with a thickness of 100 µm and a width of 1 µm, covered by 20 nm large gold pads, separated by two electrolyte contacts of the same length. The gold and electrolyte phases are separated by 1 nm of insulating vacuum to allow charge transfer only through the Si/Electrolyte and Si/Au interfaces. All the electrolyte contacts are short-circuited, so at the same potential, identically for the all gold pads. Silicon is doped n-type at a level of 3×10^{15} cm^{-3}. The work functions of gold and the electrolyte are taken at $W_{Au} = 5.5$ eV (Hölzl and Schulte, 1979) and $W_{El} = 4.5$ eV (determined in our experimental conditions, *cf.* (Torralba et al., 2016) and its supplementary information). The Fermi level is set at 0 eV at the equilibrium. To mimic the MACE process, a positive polarization can be applied between gold and the electrolyte.

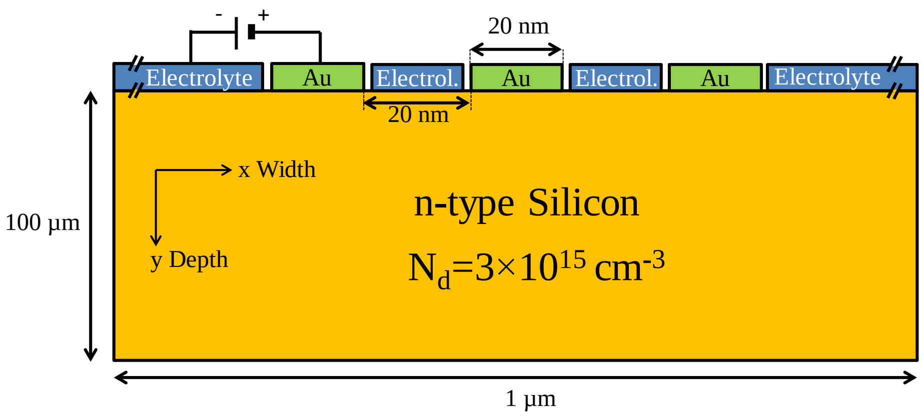

Figure D. Scheme of the device used for the band bending modeling of the Au/Si/electrolyte junctions at equilibrium and under anodic polarization of the gold pads.

Surface porosity calculation. In this work, the 2D surface porosity is the area fraction of voids (pore openings) measured on a SEM plane view image of the metal nanostructure. We used ImageJ software to convert the grey scale images in black and white images (conversion at a certain "neutral" threshold) corresponding to pores and plain gold, respectively. One can refer to the work of Seker et al. (2009, Nanoporous Gold: Fabrication, Characterization, and Applications. Materials 2, 2188. doi:10.3390/ma2042188.) for a discussion on how to determine the threshold and the uncertainty regarding the porosity value (page 2208 and 2209).
2D surface porosity might not be considered as a parameter characterizing any kind of porous material but as convenient way to compare samples with the same type of nanostructure through straightforward SEM imaging.

2. "Ancient" experiment as proof of concept

The use of nanoporous metal electrode to imprint patterns in silicon is a new concept. However, the well-known formation of silicon nanowires by MACE in HF-$AgNO_3$ medium, as initially developed by (Peng et al., 2002) is actually operating on the same principle.

Figure E shows a typical experiment of formation of silicon nanowires by MACE in HF-$AgNO_3$ medium (1 cm^2 wafer) that we conducted in the presence of HF at 1 mol L^{-1} and $AgNO_3$ at

15 10^{-3} mol L^{-1} for 1 hour. As can be seen, MACE elapses with the formation of a silver foam around the sample (held by tweezers in the picture given in Figure E-1). Although this foam has macroscopic dimensions (volume in the order of cm^2), it is constituted by silver dendrites with a diameter of about 100 nm. The base of this dendritic network is in contact with the surface of the silicon substrate (i.e. where silicon dissolution takes place), as shown by the SEM images of Figure E-2 at the beginning (left) and after a long etching time (right).

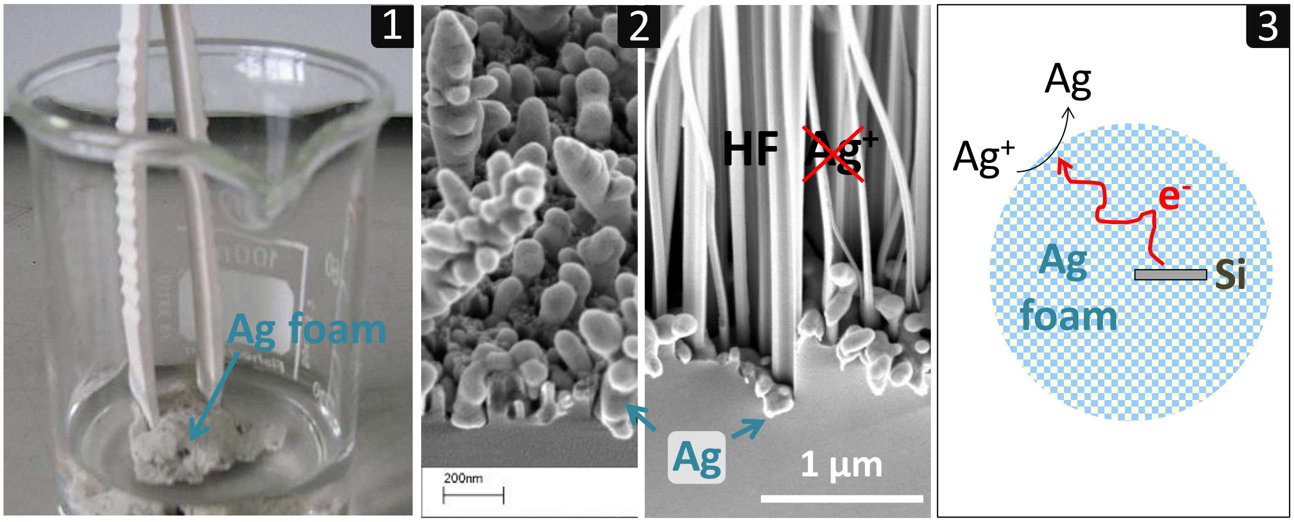

Figure E. Silicon nanowire formation in HF-$AgNO_3$: (1) silver foam formed during the experiment; (2) SEM images of the silver network in contact with silicon at the beginning of etching (left) and at the end of etching when silicon nanowires are formed (right); (3) scheme of the working principle of etching.

The extremely localized dissolution of silicon around silver results in the formation of nanowires, as shown in the SEM image (Figure E-2, right). The shape of the nanowires is smooth and precisely preserved over several tens of micrometers, which implies that the silver network is not altered during its progressive imprinting, i.e. there are no more Ag+ ions inside the foam, they are only present outside. For this process to occur, electrons from oxidized silicon atoms must be collected at the base of the dendrite network, transported through the silver foam and reduce Ag+ ions in the solution (dendritic growth by silver deposition), as shown in Figure E-3.

This well-known experiment thus demonstrates the possibility of etching silicon on a macroscopic scale with a nanoporous metal tool (silver foam) allowing both electrolyte diffusion and MACE on contact areas of sub-micrometric dimensions.

3. Electrochemical contact etching set-up

Figure D gives a scheme and a photo of the electrochemical set-up used for the electrochemical contact etching.

This is a provisional file, not the final typeset article

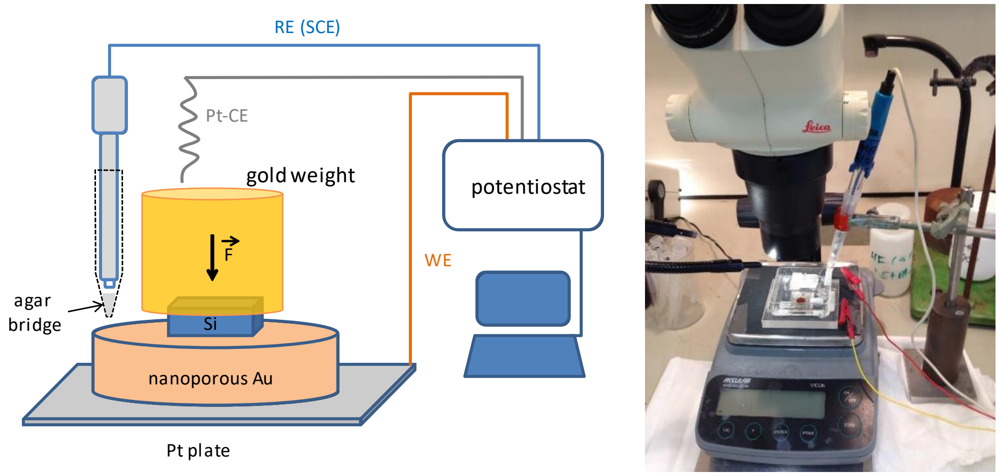

Figure F. scheme of the electrochemical set-up used for the electrochemical contact etching together with a photo (without silicon sample).

4. Contact etching data reported in Table 2 and 3

On the average, we could use 5 times np-Au electrodes with pyramids and 15 times those with lines. With pyramids, "damaged tips" is the main reason for overruling an electrode. The second pattern is logically more robust, the damages originating more from handling. For each electrode, these numbers represent all the experiments done whatever the result in terms of imprinting.

To calculate apparent etch rate values, we counted only the "successful experiments", i.e. we ruled out experiments where there had been obviously a problem, like for instance a foreign element intercalated between the silicon substrate and the electrode or if the silicon sample was placed at the level of a damaged area on the np-Au electrode. In these cases, imprinting can result in: nothing except some defects on the surface, very small etched area, usually close to the substrate edges, twisted and non-uniform patterns, etc.

For each etching conditions, 3 to 4 experiments were used to calculate an average value of the apparent etch rates given in Table 2 and 3. The maximum deviation from the average value is contained between 10 to 20 %.

5. SEM observations in cross section after contact etching of inverted pyramids in HF-H_2O_2.

The SEM images of Figure G correspond to a n-type silicon substrate chemically etch in HF-H_2O_2 with a coarse np-Au electrode for 20 min. They show that there is a porous silicon layer everywhere on the surface with a thickness of 50 nm at the bottom of an inverted pyramid and that the top of walls at the intersection of two pyramids are rounded significantly.

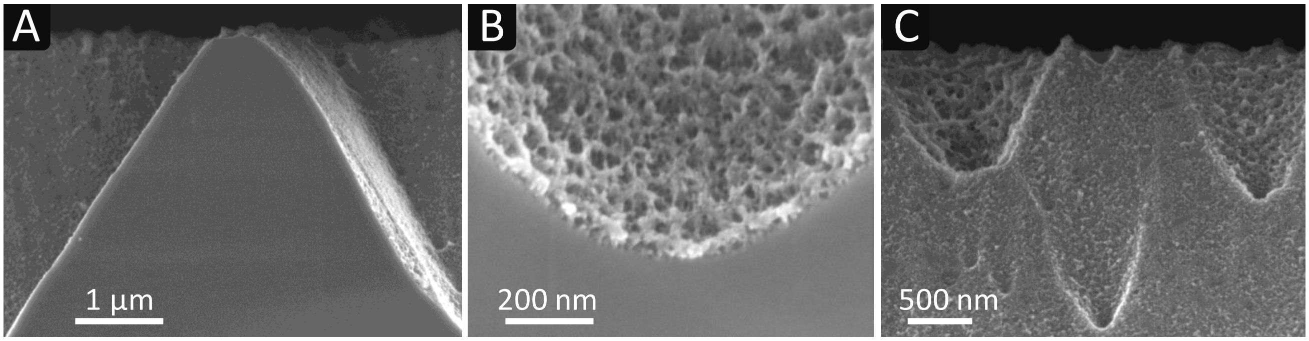

Figure G. SEM images in cross section of a silicon surface after imprinting inverted pyramids in HF-H_2O_2 with a coarse np-Au electrode. A: section of a wall between 2 inverted pyramids; B: close to the bottom of an inverted pyramid; C: pits on a wall. Electrolyte: HF 5 mol L^{-1} - H_2O_2 1 mol L^{-1}, with 2 vol.% EtOH. Etch time 20 min.

References

Hölzl, J., and Schulte, F. K. (1979). *Solid Surface Physics*. Berlin Heidelberg: Springer-Verlag Available at: //www.springer.com/us/book/9783662158098 [Accessed November 26, 2018].

Peng, K.-Q., Yan, Y.-J., Gao, S.-P., and Zhu, J. (2002). Synthesis of large-area silicon nanowire arrays via self-assembling nanoelectrochemistry. *Adv. Mater.* 14, 1164. Available at: http://atmsp.whut.edu.cn/resource/pdf/5767.pdf [Accessed January 23, 2015].

Torralba, E., Halbwax, M., El Assimi, T., Fouchier, M., Magnin, V., Harari, J., et al. (2017). 3D patterning of silicon by contact etching with anodically biased nanoporous gold electrodes. *Electrochem. Commun.* 76, 79–82. doi:10.1016/j.elecom.2017.01.014.

Torralba, E., Le Gall, S., Lachaume, R., Magnin, V., Harari, J., Halbwax, M., et al. (2016). Tunable Surface Structuration of Silicon by Metal Assisted Chemical Etching with Pt Nanoparticles under Electrochemical Bias. *ACS Appl. Mater. Interfaces* 8, 31375–31384. doi:10.1021/acsami.6b09036.

This is a provisional file, not the final typeset article
